# The adolescent with obesity: what perspectives for treatment?

**DOI:** 10.1186/s13052-022-01205-w

**Published:** 2022-01-15

**Authors:** Antonio Nicolucci, Claudio Maffeis

**Affiliations:** 1 Center for Outcomes Research and Clinical Epidemiology, CORESEARCH SRL, Corso Umberto I, 65122 Pescara, Italy; 2 Department of Surgery, Dentistry, Pediatrics and Gynecology, Section of Pediatric Diabetes and Metabolism, University and Azienda Ospedaliera Universitaria Integrata of Verona, 37126 Verona, Italy

**Keywords:** Obesity, Adolescence, Cardiometabolic risk factors, Treatment, GLP1 receptor agonists

## Abstract

The dramatic increase in overweight and obesity among children and adolescents has become a major public health problem. Obesity in children and young adults is associated with an increased prevalence of cardiometabolic risk factors. Obesity during adolescence represents a strong predictor of obesity and higher mortality in adulthood. Due to the serious implications of obesity in adolescents, effective treatments are urgently needed. Lifestyle interventions represent the recommended therapy. Nevertheless, real world data show that the majority of adolescents do not achieve weight loss in the long term, and are reluctant to participate in lifestyle interventions. Pharmacological treatment is recommended if a formal lifestyle modification program fails to limit weight gain or to improve comorbidities. However, until 2020 the European Medicines Agency (EMA) had not approved any pharmacotherapeutic agents for obesity in pediatric patients. On April 2021, EMA has authorized the use of Liraglutide, a glucagon-like peptide (GLP)-1 analog, for the treatment of obesity in adolescents (12–17 years). The efficacy and safety of Liraglutide were demonstrated in a randomized, double-blind trial, enrolling 251 adolescents. After 56 weeks, a reduction in BMI of at least 5% was observed in 43.3% of participants in the liraglutide group vs. 18.7% in the placebo group, and a reduction in BMI of at least 10% was observed in 26.1 and 8.1%, respectively. Gastrointestinal events were the events most frequently reported with liraglutide. Bariatric surgery represents another effective treatment for adolescents with severe obesity, with sustained benefits on weight loss and cardiometabolic risk factors. However, long-term safety and effectiveness data in adolescents are still scarce. Risks of bariatric surgery include the need for additional abdominal surgical procedures and specific micronutrient deficiencies. Hopefully, new pharmacological treatments in addition to lifestyle interventions will offer more chances of success.

## Introduction

Adolescence is a peculiar phase of life due to the rapid physical growth, with changes of body composition and sexual and psychologic maturation. During this delicate period, compliance to treatment of chronic diseases is modest, so that the efficacy of therapy is frequently discouraging. Also obesity, the most common chronic disease in adolescents, shows this trend. Based on this evidence, new treatments are desirable. This review reports an update of available treatments for overweight and obesity in adolescents.

### Overweight and obesity in adolescence

Over the past decades, the dramatic increase in overweight and obesity among children and adolescents has become a major public health problem, which has now reached epidemic dimensions. Global age-standardized prevalence of obesity in the 5–19 years range increased from 0.7% in 1975 to 5.6% in 2016 in girls, and from 0.9% in 1975 to 7.8% in 2016 in boys [1]. During 40 years, there was a 10-fold increase in the number of girls with obesity (from 5 million in 1975 to 50 million in 2016), and a 12-fold increase in the number of boys with obesity (from 6 million in 1975 to 74 million in 2016) [1].

According to the WHO European Childhood Obesity Surveillance Initiative (COSI), one in three children aged 6–9 years were overweight or obese in Europe in 2016–2017 [2]. Among boys, the highest prevalence of obesity was found in Cyprus and Italy (21%), followed by Spain and Greece (20%), while among girls the highest prevalence was found in Cyprus (19%) and Spain (17%), followed by Malta (15%) and Greece (14%).

A systematic review and meta-analysis assessed the prevalence trends in measured overweight and obesity among 477,620 children aged 2 to 13 years across Europe from 1999 to 2016 [3]. The combined prevalence of overweight and obesity in the Iberian region tended to decrease from 30.3 to 25.6%, while it increased in the Mediterranean region from 22.9 to 25.0%. No substantial changes were observed in Atlantic Europe or Central Europe, where the overweight and obesity prevalence changed from 18.3 to 19.3% and from 15.8 to 15.3%, respectively. The increasing prevalence of overweight and obesity in the Mediterranean region is worrisome. As an example, in Italy the prevalence of overweight among children aged 7–13 years rose from 28.2 to 35.2%, while the prevalence of obesity increased from 7.0 to 12.2% [3]. However, more recent COSI data suggest a decrease in the prevalence of overweight and obesity among children in Italy [4]. While in 2008–2009 the prevalence of overweight and obesity were 23.2 and 12.0%, respectively, in 2019 the prevalence decreased to 20.4% for overweight and 9.4% for obesity (2.4% with severe obesity).

The stabilization at high rates of obesity in children and adolescents in industrialized countries and the still increasing trend in developing countries represent a major social, clinical and economical concern, in the light of the immediate and long-term consequences of this condition.

### Obesity in adolescence and cardiometabolic risk factors

More than two-thirds of obese adolescents have at least one biochemical or clinical cardiovascular risk factor and over one quarter have more than two [5]. The association of obesity in children and young adults and an increased prevalence of cardiometabolic risk factors is well known [6–8].

A meta-analysis including 63 studies of 49,220 subjects aged 5 to 15 documented a worsening of cardiovascular risk factors in overweight and obese participants. Compared with normal weight children, systolic blood pressure was higher by 4.54 mmHg in overweight children, and by 7.49 mmHg in obese children. Similar associations were found in diastolic and 24 ambulatory systolic blood pressure. Obesity adversely affected blood lipid profile: total cholesterol and triglycerides were 0.15 mmol/L and 0.26 mmol/L higher in obese children, respectively. Fasting insulin and insulin resistance were also significantly higher in obese participants. Obese children had a significant increase in left ventricular mass of 19.12 g compared with normal weight children [9].

Elevated uric acid levels, which are frequently associated with the other traits of metabolic syndrome, are also commonly found in obese children/adolescents [10–13].

In a population of 2405 children aged 6 to 12 years, elevated levels of uric acid were found in 14.5% of normal weight participants, 28.3% of those overweight, and 43.6% of those obese [14].

High uric acid levels are also associated with reduced estimated glomerular filtration rate (eGFR) and non-alcoholic fatty liver disease (NAFLD) in children and adolescents with overweight or obesity. In a study involving 2565 young people (age 5–18 years), eGFR was calculated using the Schwartz’s bed-side formula and reduced eGFR was defined by a value < 90 mL/min/1.73 m^2^. High uric **acid** was defined as ≥75th percentile by sex in children and adolescents. Young people with high levels of uric acid had an odds ratio of 2.11 (95%CI 1.43–3.11) for reduced eGFR, an OR of 2.82 (2.26–3.45) for NAFLD; and an OR of 5.04 (3.45–7.39) for both conditions, independently of major confounders [15].

An association between elevated uric acid and risk of hypertension and diabetic kidney disease has also been described in obese adolescents with type 2 diabetes [16].

Furthermore, adolescents with obesity and the metabolic syndrome often have an increased carotid intima–media thickness, an established risk factor for cardio-cerebrovascular disease [17, 18]. Childhood obesity is also associated with arterial stiffness, one of the earliest detectable measures of vascular damage. In a meta-analysis of 15 case-control studies including 2237 children/adolescents, a significant effect of obesity on pulse wave velocity, carotid β-stiffness index, and aortic β stiffness index were documented [19].

Obesity is frequently associated with non-alcoholic fatty liver disease (NAFLD), the most common chronic liver disease in children and adolescents. NAFLD is a spectrum of progressive liver disease that spans from simple steatosis, to non-alcoholic steatohepatitis (NASH), advanced fibrosis and, ultimately, cirrhosis [20]. NAFLD also has serious health consequences outside of the liver, being strongly associated with an increased risk of cardiovascular disease and abnormal glucose tolerance [21–25].

Recently, it has been proposed to replace the term NAFLD with metabolic dysfunction-associated fatty liver disease (MAFLD). While the exclusion of other chronic liver diseases including excess alcohol intake has until now been used to establish a diagnosis of NAFLD, positive criteria have been defined for the diagnosis of MAFLD based on evidence of hepatic steatosis, in addition to one of the following three criteria, namely overweight/obesity, presence of type 2 diabetes mellitus, or evidence of metabolic dysregulation [26].

A meta-analysis on 76 independent study populations involving children aged 1–19 years documented a pooled mean prevalence of NAFLD in participants from general population studies of 7.6% (95%CI: 5.5 to 10.3%), while the prevalence reached 34.2% (95% CI: 27.8 to 41.2%) in studies based on child obesity clinics. The prevalence was higher in males compared with females and increased incrementally with greater BMI [27].. Another meta-analysis of 5 studies that assessed NAFLD by ultrasound (5305 participants aged 5 to 18 years) suggested that the prevalence of NAFLD was 26 times greater for those with obesity relative to those with a healthy weight (prevalence ratio of 26.1; 95% CI, 9.4–72.2) and 6 times greater for those who were overweight (prevalence ratio of 6.1; 95% CI, 3.3–11.2) [14].

### Obesity in adolescence: a high risk of persistence into adulthood

Obesity in childhood predicts the presence of obesity during adolescence, which in turn represents a strong predictor of obesity in adulthood.

Data from a cohort of 532 adolescents from North Norway showed that children who were overweight/obese at 5–7 years of age had increased odds of being overweight/obese at 15–17 years of age, compared to thin/normal weight children (crude odds ratio: 11.1, 95% confidence interval: 6.4–19.2). Six out of 10 children who were overweight/obese at 5–7 years of age were overweight/obese at 15–17 years of age [28].

A cohort of 215 obese Italian children aged 10.5 (±2.4) years had a follow-up examination of height and weight fourteen (±5) years later, which showed persistency of obesity (BMI > 30) in 36% of participants, whereas 32% were overweight (25 < BMI < 30), and 32% normal weight (20 < BMI < 25) [29].

In a study conducted in United States, after adjustment for parental obesity, the odds ratios for obesity in adulthood associated with childhood obesity ranged from 1.3 for obesity at 1 or 2 years of age to 17.5 for obesity at 15 to 17 years of age [30].

In Norwegian health surveys during 1963–1999, height and weight were measured for 128,121 persons in a standardized way both in adolescence (age 14–19 years) and 10 or more years later. The odds ratio of obesity (BMI ≥30) in adulthood increased steadily with BMI in adolescence, up to 16 for very high BMI [31].

In a US nationally representative cohort of 8834 individuals followed from adolescence through adulthood, obese adolescents were 16 times (HR = 16.0; 95% CI, 12.4, 20.5) more likely to develop severe obesity in young adulthood than normal weight or overweight adolescents [32].

In a systematic review and meta-analysis, obese children and adolescents were around five times more likely to be obese in adulthood than those who were not obese. About 55% of obese children were also obese in adolescence, around 80% of obese adolescents were still obese in adulthood and around 70% were obese over age 30 [33].

### Obesity in adolescence and adult morbidity and mortality

Obesity during childhood and adolescence predicts higher adult mortality. In a cohort of 4857 American Indian children without diabetes (mean age, 11.3 years), rates of death from endogenous causes among children in the highest quartile of BMI were more than double those among children in the lowest BMI quartile (incidence-rate ratio, 2.30; 95% CI, 1.46 to 3.62) [34].

In the Norwegian study cited above, very high adolescent BMI was associated with 30–40% higher adult mortality compared with medium BMI [31]..

A study on 2.3 million Israeli adolescents examined the association between BMI in late adolescence and death from cardiovascular causes in adulthood. On multivariable analysis, hazard ratios in the obese group (≥95th percentile for BMI), as compared with the reference group in the 5th to 24th percentiles, were 4.9 (95%CI, 3.9 to 6.1) for death from coronary heart disease, 2.6 (95% CI, 1.7 to 4.1) for death from stroke, 2.1 (95% CI, 1.5 to 2.9) for sudden death, and 3.5 (95% CI, 2.9 to 4.1) for death from total cardiovascular causes [35].. Of interest, the study also showed that even mild overweight during adolescence (BMI above the 50th percentile) was associated with increased risk of death from cardiovascular causes later in life.

In a cohort of 276,835 Danish schoolchildren, CHD events were ascertained by linkage to national registers. The risk of any CHD event, a nonfatal event, and a fatal event among adults was positively associated with BMI at 7 to 13 years of age for boys and 10 to 13 years of age for girls. Furthermore, the risk increased as the age of the child increased [36].

Obesity during adolescence also increases the risk of developing type 2 diabetes (T2D) and cardiovascular disease in adulthood.

In a Danish cohort of 292,827 individuals aged 7–13 years, above-average BMIs were positively associated with T2D in adult life. These associations were stronger in women than men, in younger compared with older generations, and at younger adult ages at diagnosis [37].

In a prospective study, 37,674 apparently healthy Israeli men aged 17 years were followed for incident angiography-proven coronary heart disease and diabetes. Elevated adolescent BMI was a significant predictor of both diabetes (HR for the highest vs. the lowest decile, 2.76; 95% CI, 2.11 to 3.58) and angiography-proven coronary heart disease (HR 5.43; 95% CI, 2.77 to 10.62) [38].

A systematic review and meta-analysis showed that among children aged 12 to 18 years there was a positive and statistically significant association between a one SD increase in BMI and adult diabetes (OR 1.70; 95% CI 1.30–2.22) and coronary heart disease (OR 1.30; 95% CI 1.16–1.47) [39].

A study involving 62,565 Danish men demonstrated that childhood overweight at 7 years of age was associated with increased risks of adult T2D only if it continued until puberty or later ages. Of note, the study also showed that men who had had remission of overweight before the age of 13 years had a risk of having T2D diagnosed at 30 to 60 years of age that was similar to that among men who had never been overweight [40].

More recently, a nationwide, population-based study evaluated 1,462,362 Israeli adolescents (59% men, mean age 17.4 years) during 1996–2016. Data were linked to the National Diabetes Registry. In a model adjusted for sociodemographic variables, the hazard ratios for diabetes diagnosis were 1.7 (95% CI 1.4–2.0), 2.8 (2.3–3.5), 5.8 (4.9–6.9), 13.4 (11.5–15.7), and 25.8 (21.0–31.6) among men in the 50th–74th percentile, 75th–84th percentile, overweight, mild obesity, and severe obesity groups, respectively, and 2.2 (1.6–2.9), 3.4 (2.5–4.6), 10.6 (8.3–13.6), 21.1 (16.0–27.8), and 44.7 (32.4–61.5), respectively, in women. The fractions of adult-onset T2D attributable to high BMI (≥85th percentile) at adolescence were 56.9% in men and 61.1% in women [41].

### Treatment of obesity in adolescence

#### Lifestyle interventions

Due to the serious implications of obesity in adolescents, effective treatments are urgently needed [42]. Supported by the evidence deriving from randomized trials and meta-analyses, lifestyle interventions represent the recommended therapy for adolescents with obesity [43–46]. In addition to the positive impact on weight loss, lifestyle interventions also produce a reduction in blood pressure, blood glucose, and insulin resistance, while the evidence of benefit on lipid profile is inconclusive [43, 46].

Furthermore, a meta-analysis of 19 studies that had evaluated 923 subjects aged 6–18 years showed that lifestyle intervention usually including aerobic exercise and diet produced a benefit on aminotransferase levels. Lifestyle changes also had a significant impact on steatosis, reducing the risk by 61% [47].

However, a very large, real world German study involving over 21,000 children and adolescents with obesity, showed that the majority of the participants did not achieve weight loss in the long term [48]; furthermore, over 90% of adolescents with obesity were lost to follow-up from medical care during two years of follow-up.

Furthermore, severely obese adolescents are reluctant to participate in lifestyle interventions, and response to behavioral treatment is generally limited and confined to the short term [49, 50].

The combination of diet and exercise also play an important role for the prevention of obesity among children and adolescents. A comprehensive metanalysis of studies targeting obesity prevention suggested that a combination of diet and exercise might reduce the BMI z-score (Mean Difference [MD]: -0.12; 95% CI: − 0.18 to − 0.06; 32 studies; 33,039 participants), BMI (MD: − 0.41 kg/m^2^; 95% CI: − 0.60 to − 0.21; 35 studies; 47,499 participants), and body weight (MD: -1.59; 95% CI: − 2.95 to − 0.23; 17 studies; 35,023 participants) **[**45**]**. However, prevention efforts often fail to significantly impact the weight status of children and adolescents, suggesting the need for multifactorial approaches and the involvement of different stakeholders (families, schools, policy-makers) in the decision-making process about intervention strategies to be implemented.

#### Pharmacological treatment

The 2017 Endocrine Society Guidelines suggest pharmacological treatment for adolescents, if a formal lifestyle modification program fails to limit weight gain or to improve comorbidities [51]. However, the US Food and Drug Administration has approved only two medications for the treatment of obesity in adolescents: orlistat, a lipase inhibitor, for long-term use (for ages ≥12 years) and phentermine, a norepinephrine reuptake inhibitor, for short-term use (for ages ≥17 years) [52]. Until 2020, the European Medicines Agency (EMA) had not approved any pharmacotherapeutic agents for obesity in pediatric patients. Table 1 provides a summary of the profile of anti-obesity drugs approved for obesity in adolescents.

**Table 1:** Approved medications for obesity in adolescents

Medication	Indication	Dosage	Mechanism of action	Side effects	Contraindications/warnings	Weight loss in adolescents	Approved by FDA	Approved by EMA
Orlistat	Long-term management of obesity in adolescents 12 years of age and older	120 mg three times a day with meals	Reduction of the absorption of the fatty acids consumed by food through inhibition of gastrointestinal lipases	Mostly gastrointestinal (flatulence; fecal urgency/incontinence; fatty, oily stools), generally of mild-to-moderate intensity. Mineral deficiency. Rare but serious associations of hepatic and renal illness with orlistat use have been described in the product brochure.	Chronic malabsorption syndrome, cholestasis	BMI decreased by 0.55 kg/m^2^ at 12 months. A reduction in BMI of at least 5% was observed in 26.5% of participants in the orlistat group and 15.7% participants in the placebo group [52]	Yes	No
Phentermine	Short-term management of obesity in individuals > 16 years of age	From 15 mg to 37.5 mg daily	Increase in catecholamines and serotonin activity in the central nervous system resulting in appetite suppression	Increases in heart rate and blood pressure, dry mouth, insomnia, constipation, worsening anxiety, irritability	Cardiovascular disease hyperthyroidism, active drug use, glaucoma, agitated states, pregnancy	BMI reduction of 4.1% at 6 months [53]	Yes	No
Liraglutide	Treatment of obesity in adolescence (12–17 years)	Starting dose: 0,6 mg; titration up to 3 mg daily	Glucagon-like peptide (GLP)-1 analog inducing weight loss through increased insulin secretion and counteraction of glucagon secretion depending on blood glucose levels, induction of satiety by slowing gastric emptying, and suppression of appetite by acting on the parts of the central nervous system affecting food consumption	Gastrointestinal events including nausea, vomiting, and diarrhea	Reports of pancreatitis, cholelithiasis, cholecystitis. It should be used with caution in patients with thyroid diseases. Clinically significant episodes of hypoglycemia have been reported in adolescents treated with liraglutide	Liraglutide superior to placebo with regard to the change from baseline in the BMI SDS at week 56 (estimated difference, −0.22). A reduction in BMI of at least 5% was observed in 43.3% of participants in the liraglutide group and 18.7% participants in the placebo group [54]	Yes	Yes

Orlistat reduces the absorption of approximately one third of the fatty acids consumed by food through inhibition of gastrointestinal lipases. In the largest pediatric trial, children were randomized to either orlistat 120 mg or placebo three times daily over 52 weeks. At the end of the trial, BMI had decreased by 0.55 kg/m^2^ with orlistat and increased by 0.31 kg/m^2^ with placebo. Furthermore, 26.5% of children in the orlistat group had a 5% or greater decrease in BMI compared with 15.7% of those in the placebo group [52]. Of note, only 64% of participants in the control group and 65% in the orlistat group completed the trial. The most common adverse events in the orlistat group were gastrointestinal-related, generally of mild to moderate intensity [55].

Phentermine increases catecholamines and serotonin activity in the central nervous system, resulting in appetite suppression. The main studies evaluating phentermine for the treatment of obesity in adolescents were from the 1960s, with scarce safety and efficacy data reported [53]. Increased blood pressure and heart rate are common side effects [56]. The paucity of long-term data for phentermine, along with its short-term use indication represent an important limitation, considering the need for chronic treatment of obesity.

In addition to the anti-obesity drugs, Metformin is approved by the US Food Drug Administration to treat Type 2 Diabetes in children aged over 10 years. Several studies conducted in obese children and adolescents have shown a reduction in BMI after metformin therapy, compared with the effects of lifestyle interventions alone after 6 to 12 months [57]. In a meta-analysis of 38 studies including 2199 participants metformin significantly reduced BMI [weighted mean difference (WMD): − 1.07 kg/m2; 95% confidence interval (CI): − 1.43 to − 0.72], body weight (WMD: − 2.51 kg; 95% CI: − 3.14 to − 1.89), and waist circumference (WMD: − 1.93 cm; 95% CI: − 2.69 to − 1.16) **[**57**].** In addition, Metformin has been shown to improve cardiovascular risk profile and inflammatory biomarkers in obese children and adolescents [58–60].

Commonly reported side effects are usually gastrointestinal, including bloating, diarrhea, and flatus, and are not reported as serious, with a discontinuation rate due to adverse events < 5% **[**56**].**

With limited options for antiobesity pharmacotherapy in younger individuals, metformin is frequently used off-label for adolescents **[**61**].**

In December 2020, Liraglutide has been approved for the treatment of obesity in adolescence (12–17 years) by FDA. On April 2021, the Committee for Medicinal Products for Human Use (CHMP) under the European Medicines Agency (EMA) has extended the use of Liraglutide for the treatment of obesity in adolescents aged 12–17 years. Therefore, this is the first EU-approved treatment for obesity in adolescents. Liraglutide is a glucagon-like peptide (GLP)-1 analog inducing weight loss through different mechanisms: increased insulin secretion and counteraction of glucagon secretion depending on blood glucose levels, induction of satiety by slowing gastric emptying, and suppression of appetite by acting on the parts of the central nervous system affecting food consumption [62].

The efficacy and safety of Liraglutide in adolescents were demonstrated in a randomized, double-blind trial, enrolling 251 adolescents (12 to < 18 years of age) with obesity and a poor response to lifestyle therapy alone. The trial consisted of a 56-week treatment period and a 26-week follow-up period. Participants were randomly assigned (1:1) to receive either liraglutide (3.0 mg) or placebo subcutaneously once daily, in addition to lifestyle therapy [54]. Liraglutide was superior to placebo with regard to the change from baseline in the BMI standard-deviation score at week 56 (estimated difference, − 0.22; 95% CI, − 0.37 to − 0.08). A reduction in BMI of at least 5% was observed in 43.3% of participants in the liraglutide group and 18.7% participants in the placebo group, and a reduction in BMI of at least 10% was observed in 26.1 and 8.1%, respectively. A greater reduction was observed with liraglutide than with placebo for BMI (estimated difference, − 4.64 percentage points) and for body weight (estimated difference, − 4.50 kg). At week 56, there was no substantial difference between treatment groups in glycemic and cardiometabolic variables or in overall weight-related quality of life [54]. Gastrointestinal events including nausea, vomiting, and diarrhea, were the events most frequently reported with liraglutide. Adverse events leading to discontinuation of the trial treatment occurred in 13 (10.4%) participants in the liraglutide group and none in the placebo group; in 10 participants, discontinuation was due to gastrointestinal events.

#### Bariatric surgery

Bariatric surgery is an effective treatment for adolescents with severe obesity. The Teen Longitudinal Assessment of Bariatric Surgery (Teen-LABS) study reported 3-year mean BMI reductions of 29% with Roux-en-Y gastric bypass and 27% with vertical sleeve gastrectomy among individuals aged 19 years or younger [63]. The BMI reduction was largely sustained after 5-year in the Roux-en-Y gastric bypass group [63]. In a meta-analysis of 14 studies, 950 morbidly obese adolescents with a minimum of 3 years follow-up were studied. Laparoscopic roux-en-Y gastric bypass (*n* = 453) and adjustable gastric banding (*n* = 265) were the most common bariatric procedure performed. On average, patients lost 13.3 kg/m^2^ of their BMI. Among comorbidities, only diabetes mellitus resolved or improved dramatically. Of 108 readmissions, 91 led to reoperation. There was a weight regain < 5 kg/m^2^ between 5 and 6 years of follow-up [64].

A larger meta-analysis included 49 studies with 3007 adolescents. The average preoperative age ranged from 13.9 to 19.9 years. Roux-en-Y gastric bypass (*n* = 1216), laparoscopic adjustable gastric banding (*n* = 1028), and laparoscopic sleeve gastrectomy (*n* = 665) were the most common surgeries performed. At the longest follow-up (range 12–120 months), bariatric surgery led to an overall 16.43 kg/m^2^ and 31% reduction in BMI. After 12 months from surgery, there were significant improvements in glycosylated hemoglobin, fasting blood insulin, fasting blood glucose, total cholesterol, triglycerides, high-density lipoprotein cholesterol, and low-density lipoprotein cholesterol. The remission rate of dyslipidemia was 55%, 70%, and 95% at 1, 3, and > 5 years after surgery [65].

Preliminary data suggest sustained benefits after up to 9 years in terms of weight loss and very high remission rates for lipid parameters, uric acid, liver enzymes, pre-diabetes and diabetes [66].

Despite these positive findings, long-term safety and effectiveness data in patients undergoing bariatric surgery during adolescence are still scarce. Risks of bariatric surgery include the need for additional abdominal surgical procedures and specific micronutrient deficiencies [63]. Several recognized post-operative complications may require further operative procedures, particularly symptomatic gallstone disease and small bowel obstruction. The rate of reoperation in the 5 years after RYGB appears to be slightly higher in adolescents than that in adults (20–25%) [67]. However, recent improvements in operative technique and post-operative management have led to substantial reductions in the major causes of reoperation after bariatric surgery [67].

In the FABS 5+ study, low iron and ferritin levels were reported in around two-thirds of patients, while clinical anemia was present in 46% [63]. Low vitamin D levels were documented in 78% of participants. Similarly, in the AMOS study, 61% of participants had iron deficiency and 80% had vitamin D insufficiency at 5 years [68]. Among patients with poor adherence to prescribed supplements, deficiencies in vitamins.

A, B1, B6, and B12 and folate have also been described [63].

Adolescents have been shown to experience also substantial decreases in bone mineral density [69]. These findings deserve particular consideration in the light of the described increased risk of bone fractures among adults undergoing bariatric surgery [70].

## Conclusions

Obesity during childhood and adolescence represent a global health problem. Obesity in adolescence is associated with significant cardio-metabolic comorbidities and biochemical alterations, including hypertension, dyslipidemia, dysglycemia and hyperinsulinemia, hyperuricemia, MAFLD, and increased risk for Polycystic Ovary Syndrome (PCOS) in girls. Many obese adolescents remain obese until adulthood, with a markedly increase in morbidity and mortality later in life.

Addressing obesity in adolescence is therefore an important priority. Public health initiatives for primary prevention of obesity in children and adolescents remain the cornerstone to fight the continuous rise of obesity prevalence. However, despite isolated areas of improvement, no country to date has reversed its obesity epidemic [71]. To be effective, interventions at the population level require profound changes to social and cultural norms, multi-sector initiatives, including government, education, health care, marketing, and food and beverage industries, and interventions in different settings, such as schools, worksites, and.

community. Lifestyle interventions and available pharmacological treatments produce limited benefits, and most effective and safe strategies for weight reduction are urgently needed. The portfolio of available treatment options is extremely limited: bariatric surgery is only indicated for severe obesity and is not free from complications, while pharmacological treatments currently available are only a few, with limited evidence of long-term benefits. Hopefully, new pharmacological treatments to add to lifestyle interventions can offer more chances of success. Recently, the treatment with GLP1 receptor agonist showed encouraging results in adolescents with obesity and can help to reduce the clinical, social and economic burden of this condition.

## Data Availability

(not applicable)

## Abbreviations

BMI: body mass index
CHMP: Committee for Medicinal Products for Human Use
CI: confidence interval
COSI: Childhood Obesity Surveillance Initiative
eGFR: estimated glomerular filtration rate
EMA: European Medicines Agency
FDA: Food and Drug Administration
GLP: glucagon-like peptide
MAFLD: metabolic dysfunction-associated fatty liver disease
NASH: non-alcoholic steatohepatitis
NAFLD: non-alcoholic fatty liver disease
OR: odds ratio
PCOS: Polycystic Ovary Syndrome
Teen-LABS: Teen Longitudinal Assessment of Bariatric Surgery
T2D: type 2 diabetes

## References

[1] NCD Risk Factor Collaboration (NCD-RisC) (2017). Worldwide trends in body-mass index, underweight, overweight, and obesity from 1975 to 2016: a pooled analysis of 2416 population-based measurement studies in 128·9 million children, adolescents, and adults. Lancet..

[2] https://www.euro.who.int/__data/assets/pdf_file/0006/372426/WH14_COSI_factsheets_v2.pdf

[3] Garrido-Miguel M, Cavero-Redondo I, Álvarez-Bueno C, Rodríguez-Artalejo F, Moreno LA, Ruiz JR, Ahrens W, Martínez-Vizcaíno V (2019). Prevalence and trends of overweight and obesity in European children from 1999 to 2016: a systematic review and Meta-analysis. JAMA Pediatr.

[4] https://www.epicentro.iss.it/okkioallasalute/indagine-2019-dati

[5] Weiss R, Dziura J, Burgert TS, Tamborlane WV, Taksali SE, Yeckel CW, Allen K, Lopes M, Savoye M, Morrison J, Sherwin RS, Caprio S (2004). Obesity and the metabolic syndrome in children and adolescents. N Engl J Med.

[6] Skinner AC, Perrin EM, Moss LA, Skelton JA (2015). Cardiometabolic risks and severity of obesity in children and young adults. N Engl J Med.

[7] Juonala M, Magnussen C, Berenson G (2011). Childhood adiposity, adult adiposity, and cardiovascular risk factors. N Engl J Med.

[8] Gidding SS, Nehgme R, Heise C, Muscar C, Linton A, Hassink S (2004). Severe obesity associated with cardiovascular deconditioning, high prevalence of cardiovascular risk factors, diabetes mellitus/hyperinsulinemia, and respiratory compromise. J Pediatr.

[9] Friedemann C, Heneghan C, Mahtani K, Thompson M, Perera R, Ward AM (2012). Cardiovascular disease risk in healthy children and its association with body mass index: systematic review and meta-analysis. BMJ..

[10] Goli P, Riahi R, Daniali SS, Pourmirzaei M, Kelishadi R (2020). Association of serum uric acid concentration with components of pediatric metabolic syndrome: a systematic review and meta-analysis. J Res Med Sci.

[11] Di Bonito P, Valerio G, Licenziati MR, Campana G, Del Giudice EM, Di Sessa A, Morandi A, Maffeis C, Chiesa C, Pacifico L, Baroni MG, Manco M (2021). Uric acid, impaired fasting glucose and impaired glucose tolerance in youth with overweight and obesity. Nutr Metab Cardiovasc Dis.

[12] Lurbe E, Torro MI, Alvarez-Pitti J, Redon J, Borghi C, Redon P (2018). Uric acid is linked to cardiometabolic risk factors in overweight and obese youths. J Hypertens.

[13] Kong AP, Choi KC, Ho CS, Chan MH, Ozaki R, Chan CW, Chan JC (2013). Associations of uric acid and gamma-glutamyltransferase (GGT) with obesity and components of metabolic syndrome in children and adolescents. Pediatr Obes.

[14] Sharma V, Coleman S, Nixon J, Sharples L, Hamilton-Shield J, Rutter H, Bryant M (2019). A systematic review and meta-analysis estimating the population prevalence of comorbidities in children and adolescents aged 5 to 18 years. Obes Rev.

[15] Di Bonito P, Valerio G, Licenziati MR, Miraglia Del Giudice E, Baroni MG, Morandi A, Maffeis C (2020). High uric acid, reduced glomerular filtration rate and non-alcoholic fatty liver in young people with obesity. J Endocrinol Investig.

[16] Bjornstad P, Laffel L, Lynch J, El Ghormli L, Weinstock RS, Tollefsen SE, Nadeau KJ, TODAY Study Group (2019). Elevated serum uric acid is associated with greater risk for hypertension and diabetic kidney diseases in obese adolescents with type 2 diabetes: an observational analysis from the treatment options for type 2 diabetes in adolescents and youth (TODAY) study. Diabetes Care.

[17] Reinehr T, Wunsch R, Putter C, Scherag A (2013). Relationship between carotid intima-media thickness and metabolic syndrome in adolescents. J Pediatr.

[18] Hudson LD, Kinra S, Wong I, Viner RM (2017). Arterial stiffening, insulin resistance and acanthosis nigricans in a community sample of adolescents with obesity. Int J Obes.

[19] Cote AT, Phillips AA, Harris KC, Sandor GG, Panagiotopoulos C, Devlin AM (2015). Obesity and arterial stiffness in children: systematic review and meta-analysis. Arterioscler Thromb Vasc Biol.

[20] Younossi Z, Anstee QM, Marietti M, Hardy T, Henry L, Eslam M, George J, Bugianesi E (2018). Global burden of NAFLD and NASH: trends, predictions, risk factors and prevention. Nat Rev Gastroenterol Hepatol.

[21] Selvakumar PKC, Kabbany MN, Nobili V, Alkhouri N (2017). Nonalcoholic fatty liver disease in children: hepatic and extrahepatic complications. Pediatr Clin N Am.

[22] Byrne CD, Targher G (2015). NAFLD: a multisystem disease. J Hepatol.

[23] Adams LA, Anstee QM, Tilg H, Targher G (2017). Non-alcoholic fatty liver disease and its relationship with cardiovascular disease and other extra-hepatic diseases. Gut.

[24] Targher G, Lonardo A, Byrne CD (2018). Nonalcoholic fatty liver disease and chronic vascular complications of diabetes mellitus. Nat Rev Endocrinol.

[25] Nobili V, Mantovani A, Cianfarani S, Alisi A, Mosca A, Sartorelli MR, Maffeis C, Loomba R, Byrne CD, Targher G (2019). Prevalence of prediabetes and diabetes in children and adolescents with biopsy-proven non-alcoholic fatty liver disease. J Hepatol.

[26] Eslam M, Newsome PN, Sarin SK, Anstee QM, Targher G, Romero-Gomez M, Zelber-Sagi S, Wai-Sun Wong V, Dufour JF, Schattenberg JM, Kawaguchi T, Arrese M, Valenti L, Shiha G, Tiribelli C, Yki-Järvinen H, Fan JG, Grønbæk H, Yilmaz Y, Cortez-Pinto H, Oliveira CP, Bedossa P, Adams LA, Zheng MH, Fouad Y, Chan WK, Mendez-Sanchez N, Ahn SH, Castera L, Bugianesi E, Ratziu V, George J (2020). A new definition for metabolic dysfunction-associated fatty liver disease: An international expert consensus statement. J Hepatol.

[27] Anderson EL, Howe LD, Jones HE, Higgins JPT, Lawlor DA, Fraser A (2015). The prevalence of non-alcoholic fatty liver disease in children and adolescents: a systematic review and meta-analysis. PLoS One.

[28] Evensen E, Wilsgaard T, Furberg AS, Skeie G (2016). Tracking of overweight and obesity from early childhood to adolescence in a population-based cohort - the Tromsø study. Fit Futures BMC Pediatr.

[29] Maffeis C, Moghetti P, Grezzani A, Clementi M, Gaudino R, Tatò L (2002). Insulin resistance and the persistence of obesity from childhood into adulthood. J Clin Endocrinol Metab.

[30] Whitaker RC, Wright JA, Pepe MS, Seidel KD, Dietz WH (1997). Predicting obesity in young adulthood from childhood and parental obesity. N Engl J Med.

[31] Engeland A, Bjørge T, Tverdal A, Søgaard AJ (2004). Obesity in adolescence and adulthood and the risk of adult mortality. Epidemiology..

[32] Suchindran C, North KE, Popkin BM, Gordon-Larsen P, The NS (2010). Association of adolescent obesity with risk of severe obesity in adulthood. JAMA..

[33] Simmonds M, Llewellyn A, Owen CG, Woolacott N (2016). Predicting adult obesity from childhood obesity: a systematic review and meta-analysis. Obes Rev.

[34] Franks PW, Hanson RL, Knowler WC, Sievers ML, Bennett PH, Looker HC (2010). Childhood obesity, other cardiovascular risk factors, and premature death. N Engl J Med.

[35] Twig G, Yaniv G, Levine H, Leiba A, Goldberger N, Derazne E, Ben-Ami Shor D, Tzur D, Afek A, Shamiss A, Haklai Z, Kark JD (2016). Body-mass index in 2.3 million adolescents and cardiovascular death in adulthood. N Engl J Med.

[36] Baker JL, Olsen LW, Sørensen TI (2007). Childhood body-mass index and the risk of coronary heart disease in adulthood. N Engl J Med.

[37] Zimmermann E, Bjerregaard LG, Gamborg M, Vaag AA, Sørensen TIA, Baker JL (2017). Childhood body mass index and development of type 2 diabetes throughout adult life-a large-scale Danish cohort study. Obesity (Silver Spring).

[38] Tirosh A, Shai I, Afek A, Dubnov-Raz G, Ayalon N, Gordon B, Derazne E, Tzur D, Shamis A, Vinker S, Rudich A (2011). Adolescent BMI trajectory and risk of diabetes versus coronary disease. N Engl J Med.

[39] Llewellyn A, Simmonds M, Owen CG, Woolacott N (2016). Childhood obesity as a predictor of morbidity in adulthood: a systematic review and meta-analysis. Obes Rev.

[40] Bjerregaard LG, Jensen BW, Ängquist L, Osler M, Sørensen TIA, Baker JL (2018). Change in overweight from childhood to early adulthood and risk of type 2 diabetes. N Engl J Med.

[41] Twig G, Zucker I, Afek A, Cukierman-Yaffe T, Bendor CD, Derazne E, Lutski M, Shohat T, Mosenzon O, Tzur D, Pinhas-Hamiel O, Tiosano S, Raz I, Gerstein HC, Tirosh A (2020). Adolescent obesity and early-onset type 2 diabetes. Diabetes Care.

[42] Valerio G, Maffeis C, Saggese G, Ambruzzi MA, Balsamo A, Bellone S, Bergamini M, Bernasconi S, Bona G, Calcaterra V, Canali T, Caroli M, Chiarelli F, Corciulo N, Crinò A, di Bonito P, di Pietrantonio V, di Pietro M, di Sessa A, Diamanti A, Doria M, Fintini D, Franceschi R, Franzese A, Giussani M, Grugni G, Iafusco D, Iughetti L, Lamborghini A, Licenziati MR, Limauro R, Maltoni G, Manco M, Reggiani LM, Marcovecchio L, Marsciani A, del Giudice EM, Morandi A, Morino G, Moro B, Nobili V, Perrone L, Picca M, Pietrobelli A, Privitera F, Purromuto S, Ragusa L, Ricotti R, Santamaria F, Sartori C, Stilli S, Street ME, Tanas R, Trifiró G, Umano GR, Vania A, Verduci E, Zito E (2018). Diagnosis, treatment and prevention of pediatric obesity: consensus position statement of the Italian Society for Pediatric Endocrinology and Diabetology and the Italian Society of Pediatrics. Ital J Pediatr.

[43] Stoner L, Rowlands D, Morrison A, Credeur D, Hamlin M, Gaffney K, Lambrick D, Matheson A (2016). Efficacy of exercise intervention for weight loss in overweight and obese adolescents: Meta-analysis and implications. Sports Med.

[44] Kelley GA, Kelley KS, Pate RR (2017). Exercise and BMI z-score in overweight and obese children and adolescents: a systematic review and network Meta-analysis of randomized trials. J Evid Based Med.

[45] Salam RA, Padhani ZA, Das JK, Shaikh AY, Hoodbhoy Z, Jeelani SM, Lassi ZS, Bhutta ZA (2020). Effects of lifestyle modification interventions to prevent and manage child and adolescent obesity: a systematic review and Meta-analysis. Nutrients..

[46] O'Connor EA, Evans CV, Burda BU, Walsh ES, Eder M, Lozano P (2017). Screening for obesity and intervention for weight Management in Children and Adolescents: evidence report and systematic review for the US preventive services task force. JAMA..

[47] Utz-Melere M, Targa-Ferreira C, Lessa-Horta B, Epifanio M, Mouzaki M, Mattos AA (2018). Non-alcoholic fatty liver disease in children and adolescents: lifestyle change - a systematic review and Meta-analysis. Ann Hepatol.

[48] Reinehr T, Widhalm K, l'Allemand D, Wiegand S, Wabitsch M, Holl RW, APV-Wiss STudy Group and German Competence Net Obesity (2009). Two-year follow-up in 21,784 overweight children and adolescents with lifestyle intervention. Obesity (Silver Spring).

[49] Knop C, Singer V, Uysal Y, Schaefer A, Wolters B, Reinehr T (2015). Extremely obese children respond better than extremely obese adolescents to lifestyle interventions. Pediatric Obesity.

[50] Beamish AJ, Reinehr T (2017). Should bariatric surgery be performed in adolescents?. Eur J Endocrinol.

[51] Styne DM, Arslanian SA, Connor EL, Farooqi IS, Murad MH, Silverstein JH, Yanovski JA (2017). Pediatric obesity-assessment, treatment, and prevention: an Endocrine Society clinical practice guideline. J Clin Endocrinol Metab.

[52] Cardel MI, Jastreboff AM, Kelly AS (2019). Treatment of adolescent obesity in 2020. JAMA..

[53] Ryder JR, Kaizer A, Rudser KD, Gross A, Kelly AS, Fox CK (2017). Effect of phentermine on weight reduction in a pediatric weight management clinic. Int J Obes.

[54] Kelly AS, Auerbach P, Barrientos-Perez M, Gies I, Hale PM, Marcus C, Mastrandrea LD, Prabhu N, Arslanian S, NN8022–4180 Trial Investigators. A Randomized (2020). Controlled Trial of Liraglutide for Adolescents with Obesity. N Engl J Med.

[55] Chanoine JP, Hampl S, Jensen C, Boldrin M, Hauptman J (2005). Effect of orlistat on weight and body composition in obese adolescents: a randomized controlled trial. JAMA.

[56] Woodard K, Louque L, Hsia DS (2020). Medications for the treatment of obesity in adolescents. Ther Adv Endocrinol Metab.

[57] Sadeghi A, Mousavi SM, Mokhtari T, Parohan M, Milajerdi A (2020). Metformin therapy reduces obesity indices in children and adolescents: a systematic review and Meta-analysis of randomized clinical trials. Child Obes.

[58] Evia-Viscarra ML, Rodea-Montero ER, Apolinar-Jiménez E, Muñoz-Noriega N, García-Morales LM, Leaños-Pérez C, Figueroa-Barrón M, Sánchez-Fierros D, Reyes-García JG (2012). The effects of metformin on inflammatory mediators in obese adolescents with insulin resistance: controlled randomized clinical trial. J Pediatr Endocrinol Metab.

[59] Gómez-Díaz RA, Talavera JO, Pool EC, Ortiz-Navarrete FV, Solórzano-Santos F, Mondragón-González R, Valladares-Salgado A, Cruz M, Aguilar-Salinas CA, Wacher NH (2012). Metformin decreases plasma resistin concentrations in pediatric patients with impaired glucose tolerance: a placebo controlled randomized clinical trial. Metabolism..

[60] Mauras N, DelGiorno C, Hossain J, Bird K, Killen K, Merinbaum D, Weltman A, Damaso L, Balagopal P (2012). Metformin use in children with obesity and normal glucose tolerance–effects on cardiovascular markers and intrahepatic fat. J Pediatr Endocrinol Metab.

[61] Srivastava G, Fox CK, Kelly AS, Jastreboff AM, Browne AF, Browne NT, Pratt JSA, Bolling C, Michalsky MP, Cook S, Lenders CM, Apovian CM (2019). Clinical considerations regarding the use of obesity pharmacotherapy in adolescents with obesity. Obesity (Silver Spring).

[62] Van Can J, Sloth B, Jensen CB (2014). Effects of the once-daily GLP-1 analog liraglutide on gastric emptying, glycemic parameters, appetite and energy metabolism in obese, non-diabetic adults. Int J Obes.

[63] Inge TH, Courcoulas AP, Jenkins TM, Teen-LABS Consortium (2016). Weight loss and health status 3 years after bariatric surgery in adolescents. N Engl J Med.

[64] Shoar S, Mahmoudzadeh H, Naderan M, Bagheri-Hariri S, Wong C, Parizi AS, Shoar N (2017). Long-term outcome of bariatric surgery in morbidly obese adolescents: a systematic review and Meta-analysis of 950 patients with a minimum of 3 years follow-up. Obes Surg.

[65] Qi L, Guo Y, Liu CQ, Huang ZP, Sheng Y, Zou DJ (2017). Effects of bariatric surgery on glycemic and lipid metabolism, surgical complication and quality of life in adolescents with obesity: a systematic review and meta-analysis. Surg Obes Relat Dis.

[66] Elhag W, El Ansari W (2021). Durability of Cardiometabolic outcomes among adolescents after sleeve gastrectomy: first study with 9-year follow-up. Obes Surg.

[67] Chalklin CG, Ryan Harper EG, Beamish AJ (2021). Metabolic and bariatric surgery in adolescents. Curr Obes Rep.

[68] Henfridsson P, Laurenius A, Wallengren O, Beamish AJ, Dahlgren J, Flodmark CE, Marcus C, Olbers T, Gronowitz E, Ellegard L (2019). Micronutrient intake and biochemistry in adolescents adherent or nonadherent to supplements 5 years after roux-en-Y gastric bypass surgery. Surg Obes Relat Dis.

[69] Beamish AJ, Gronowitz E, Olbers T, Flodmark CE, Marcus C, Dahlgren J (2017). Body composition and bone health in adolescents after roux-en-Y gastric bypass for severe obesity. Pediatric Obes.

[70] Ahlin S, Peltonen M, Sjöholm K, Anveden Å, Jacobson P, Andersson-Assarsson JC, Taube M, Larsson I, Lohmander LS, Näslund I, Svensson PA, Carlsson LMS (2020). Fracture risk after three bariatric surgery procedures in Swedish obese subjects: up to 26 years follow-up of a controlled intervention study. J Intern Med.

[71] Roberto CA, Swinburn B, Hawkes C, Huang TTK, Costa SA, Ashe M, Zwicker L, Cawley JH, Brownell KD (2015). Patchy progress on obesity prevention: emerging examples, entrenched barriers, and new thinking. Lancet.

